# Notes and News

**Published:** 1903-12-19

**Authors:** 


### Notes and News.

Lord Iveagh's munificent gift of £50,000 to the Dublin hospitals, in commemoration of the King's visit to Ireland, has now been distributed.

Mrs. Eliza Darling has bequeathed £1,500 to the convalescent home of the Victoria Hospital for Children, and £2,000 to the Victoria Home for Invalid Children at Margate.

A new out-patients' department has been opened at the Queen Victoria Royal Infirmary, Preston. It includes, besides general waiting-hall and consulting rooms, an ophthalmic room, laboratory, dispensary, accident, surgery, and other offices.

It is very amusing to read Mr. Stephen Coleridge's letter of disgust in the *Times* of December 11th, in which he disclaims any sympathy with the gross exaggeration and unfairness of the Canine Defence League in its attack upon vivisection. He is anxious that all anti-vivisectionists should disassociate themselves from the League.

It is impossible to understand the credulity of the public in the matter of medical cures. At a recent inquest the fact was elicited that a young man who had attended the Brompton Hospital for Consumption, and who was told that his case was hopeless, afterwards placed himself in the hands of a man who was described on his card as Captain Mark Golinsky, and below his name were the words "Consumption Cure Co. Consumption cured in its last stages." Captain Golinsky laid no claim to any medical qualification.

It is proposed to create a Chair of General Physics at the Paris Faculty of Science, and to invite Professor Curie to be the first occupant.

It has been ascertained that oysters coming from Long Bay at the head of Sydney Middle Harbour are infected with typhoid. The Board of Health recommends the Government to issue an order forbidding oysters to be taken from this locality for the space of three years.

The late Miss Hamilton L. Hamilton of Bute has bequeathed £10,000 to the Glasgow Royal Infirmary, £30,000 to the Glasgow Western Infirmary, to found a Hamilton Ward, £7,500 each to the Association for the Relief of Incurables, the Edinburgh Royal Infirmary, the Victoria Infirmary, and Glasgow Hospital for Sick Children, £5,000 to the Longmore Hospital, and £2,500 to the West of Scotland Convalescent Home at Dunoon.

Sir William Anson, when speaking at Preston, expressed an opinion that bad cooking was one of the factors which led to drunkenness, and urged that cookery should be made an important item of elementary education. We cordially agree with Sir William Anson, and go further and believe that cooking has much to do with the health of the poorer classes, especially in respect to the children. The subject is worthy of a consideration it has never yet received.

The Borough Council of Camberwell have passed a resolution welcoming the decision of the authorities of King's College Hospital to remove the institution to South London, and pledging themselves to do everything in their power to further the appeal for funds. It is much to be hoped that local support will be forthcoming sufficient in amount to defray the cost of one block or wing of the new hospital. The Drapers' Company have voted the sum of £5,000 to the Removal Fund, conditionally upon the balance of the £300,000 required being raised by voluntary contributions within twelve calendar months.

We are informed by the authorities of the Derbyshire Royal Infirmary that during the past week two important deputations from other towns have visited the institution for the purpose of inspecting the buildings and arrangements for the comfort of the patients generally. One deputation consisted of 15 members of the honorary medical staff of King's College Hospital, London, accompanied by the architect, the principal (Rev. Arthur C. Headlam), and the matron (Miss Monk); the other was the chairman and members of the committee and honorary medical staff of the Children's Infirmary, Liverpool, and the general superintendent of the Liverpool Royal Infirmary. Both King's College and Liverpool are proposing to build new hospitals, and the visitors, who spent nearly five hours in the institution, obtained much valuable information, and expressed to the members of the honorary and resident medical staffs, and to the secretary-superintendent, and matron, who met and conducted them through the infirmary, the gratification their visit gave them, several stating that it was the best hospital they had ever inspected.

